# Enhancing the CO_2_ Electroreduction of Fe/Ni‐Pentlandite Catalysts by S/Se Exchange

**DOI:** 10.1002/chem.202001289

**Published:** 2020-07-08

**Authors:** Kevinjeorjios Pellumbi, Mathias Smialkowski, Daniel Siegmund, Ulf‐Peter Apfel

**Affiliations:** ^1^ Inorganic Chemistry I Ruhr University Bochum Universitätsstraße 150 44801 Bochum Germany; ^2^ Division of Energy Department Think Tank/Electrosynthesis Fraunhofer UMSICHT Osterfelderstraße 3 46047 Oberhausen Germany

**Keywords:** CO_2_ reduction, electrocatalysis, hydrogen, pentlandite, sulfoselenides

## Abstract

The electrochemical reduction of CO_2_ is an attractive strategy towards the mitigation of environmental pollution and production of bulk chemicals as well as fuels by renewables. The bimetallic sulfide Fe_4.5_Ni_4.5_S_8_ (pentlandite) was recently reported as a cheap and robust catalyst for electrochemical water splitting, as well as for CO_2_ reduction with a solvent‐dependent product selectivity. Inspired by numerous reports on monometallic sulfoselenides and selenides revealing higher catalytic activity for the CO_2_ reduction reaction (CO_2_RR) than their sulfide counterparts, the authors investigated the influence of stepwise S/Se exchange in seleno‐pentlandites Fe_4.5_Ni_4.5_S_8‐Y_Se_Y_ (Y=1–5) and their ability to act as CO_2_ reducing catalysts. It is demonstrated that the incorporation of higher equivalents of selenium favors the CO_2_RR with Fe_4.5_Ni_4.5_S_4_Se_4_ revealing the highest activity for CO formation. Under galvanostatic conditions in acetonitrile, Fe_4.5_Ni_4.5_S_4_Se_4_ generates CO with a Faradaic Efficiency close to 100 % at applied current densities of −50 mA cm^−2^ and −100 mA cm^−2^. This work offers insight into the tunability of the pentlandite based electrocatalysts for the CO_2_ reduction reaction.

## Graphical abstract

**The electrocatalytic CO_2_RR capabilities** of the bifunctional pentlandite system were varied by gradual exchange of sulfur by selenium. Furthermore, the ratio of H_2_ to CO could be further improved by regulation of the water content of the reactor system.

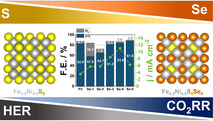

## Introduction

The continuous consumption of fossil fuels by modern society leads to rising atmospheric CO_2_ concentrations, which are linked to severe detrimental effects to the environment^1^ and human health.^2^ Consequently, achieving a closed carbon cycle economy, that is, creating processes that do not cause a net increase in CO_2_ emissions, is considered an important strategy to enhance global sustainability.^3^ As an integral part of this approach, CO_2_ electroreduction aims at using carbon dioxide as a C1 building block for commodity chemicals and fuels. Additionally, electrochemical processes are ideal to counterbalance the fluctuating nature of renewable energy sources (e.g., wind or solar power) allowing to continuously store excess energy within chemical bonds.^4^ However, major challenges, such as a low mass transport and solubility of CO_2_ in commonly employed aqueous electrolytes, and the reduced product selectivity, partially caused by the competing production of dihydrogen, still hamper large‐scale implementation.^5^ Furthermore, the most efficient catalysts for these transformations are made from expensive and unsustainable metals such as gold and silver which mainly yield CO, from indium and tin mainly producing formate, and from copper for obtaining C−C coupling products.^6^ Recently, interesting alternatives have been suggested using layered transition metal chalcogenides on molybdenum basis including Ni functionalization.^7^

Along this line, the Fe/Ni‐pentlandite (Fe_4.5_Ni_4.5_S_8_, **Pn**), a bioinspired mineral analogue of nickel containing CODH enzymes,^8^ has been shown by our group to be a robust and efficient catalyst for the CO_2_ reduction reaction (CO_2_RR).^9^

In organic electrolytes featuring a rather high solubility of CO_2_, that is, high CO_2_ mass transport to the electrode compared to aqueous electrolytes, **Pn** showed a solvent dependent selectivity for CO_2_RR products, such as CO and CH_4_. Likewise, hydrogen generation and CO_2_ reduction can be understood as two competing reactions where the catholyte proton concentration is a decisive factor in modulating the ratio between these two reduction pathways. Notably, the use of organic solvents allows to tightly adjust the proton concentration of the catholyte and through stepwise variation of the catholytes water content the production of syngas in controllable H_2_/CO ratios is conceivable. Although surrounding parameters, such as the electrolyte,^10^ pressure^11^ and temperature,^12^ can influence the CO_2_RR outcome, differences between the rich compositional varieties of pentlandites on the CO_2_ reduction were never tested before.

While numerous reports addressed the influence of homologous chalcogenide exchange, in particular substitution of sulfur for selenium, for the hydrogen evolution reaction (HER), the concept of S/Se‐exchange is only rudimentary explored for CO_2_RR and has yielded contradicting results. For example, and contrary to MoS_2_, an increased CO_2_RR activity was observed by MoSSe for the formation of CO.^13^ Conversely, a decreased CO_2_RR efficiency was observed for CO generation by CdSe in comparison to CdS.^14^

Thus, we herein set out to assess the effect of S/Se exchange on the CO_2_RR activity in Fe_4.5_Ni_4.5_S_8_ pentlandite. This project is further motivated by our recent observation that a stepwise S/Se exchange suppresses the HER activity of **Pn** most likely due to an increase of the interatomic metal distances among other effects.^15^ A high selenium content beyond Fe_4.5_Ni_4.5_S_7_Se should thus inhibit hydrogen evolution and favorable CO_2_RR‐properties due to suppression of its main competitor reaction can be expected. We herein show that the CO_2_ reduction activity of **Pn** can be enhanced by introducing high stoichiometric amounts of selenium to obtain high Faradaic efficiency (F.E.) for CO at high current densities in organic catholytes.

## Results and Discussion

In accordance to the previously established synthetic boundaries of Se‐containing pentlandites, our investigations focus on the reported seleno‐pentlandites Fe_4.5_Ni_4.5_S_8‐Y_Se_Y_ (Y=1–5, **Se‐1**, **Se‐2**, **Se‐3**, **Se‐4**, **Se‐5**) with integer equivalents of selenium as well as the selenium‐free pentlandite (**Pn**) for comparative purposes. The respective materials were synthesized by high‐temperature solid‐state synthesis from the respective elemental mixtures according to previously published reports.^15^, ^16^ The characterization of the materials obtained, especially powder‐XRD, single‐crystal XRD, DSC as well as XPS correspond to the results previously reported by our group.^15^ The as synthesized rock material was investigated for its CO_2_RR performance in the form of polished pellet electrodes encapsulated in a Teflon housing (0.071 cm^−2^) using a three‐electrode H‐type cell setup in a continuous CO_2_ flow operation mode. CO_2_‐saturated acetonitrile with low (24×10^−3^ mg mL^−1^, 24 ppm) and high (30 mg mL^−1^, 30 000 ppm) water content was employed as organic electrolyte to tightly control the proton concentration within our setup and with it the competing hydrogen evolution. As a precautionary measure to minimize the decomposition of acetonitrile on the anode side when employing electrolytes with low proton availability, the anolyte was continuously purged with wetted N_2_ at a flow rate of 10 mL min^−1^ during electrolysis. Throughout the manuscript we will refer to electrolytes with a low water content as “*dry*” electrolyte whereas high water electrolytes will be denoted as “*wet*”, respectively. In order to initially assess the fundamental electrochemical properties of the electrodes we investigated their behavior in *wet* and *dry* acetonitrile with the help of linear sweep voltammetry (LSV) in the range of 0 V to −2 V vs. NHE with a scan rate of 5 mV s^−1^.

**Figure 1:**
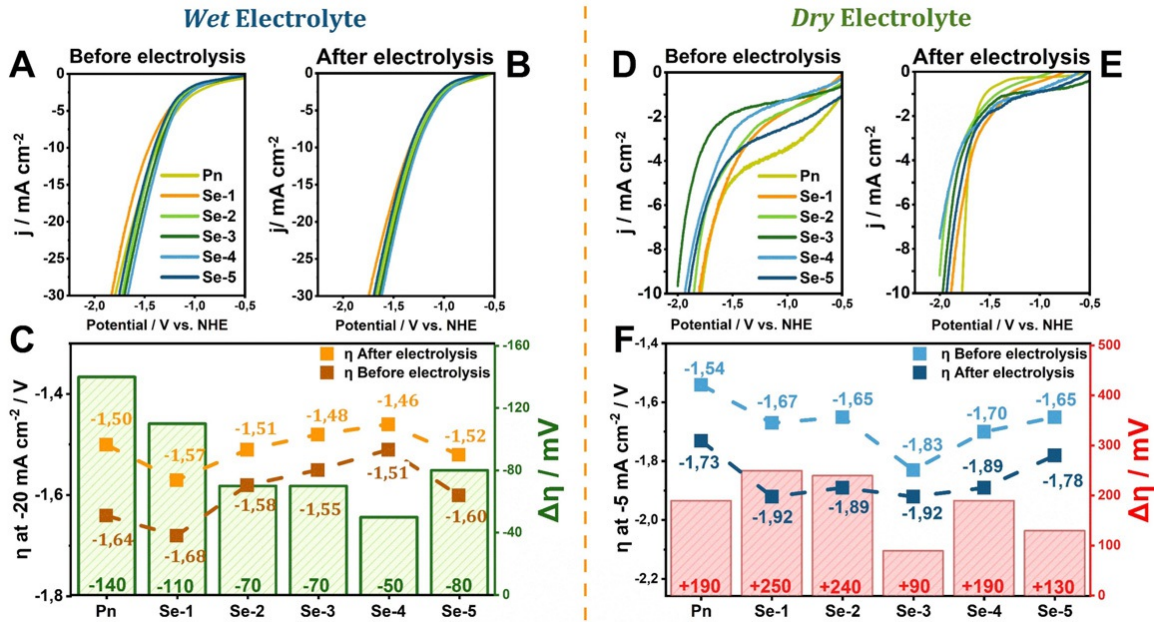
LSV curves of **Pn** to **Se‐5** before (A) and after electrolysis at −1.8 V vs. NHE (B) in *wet* electrolyte recorded at a scan rate of 5 mV s^−1^. Overpotential of **Pn** to **Se‐5** before and after electrolysis at −20 mA cm^−2^ in *wet* electrolyte (C). LSV curves of **Pn** to **Se‐5** before (D) and after electrolysis (E) at −1.8 V vs. NHE in *dry* electrolyte recorded at scan rate of 5 mV s^−1^. Overpotential of **Pn**–**Se‐5** before and after electrolysis at −5 mA cm^−2^ in the *wet* electrolyte (F).

In *wet* acetonitrile the investigated catalysts **Pn** as well as **Se‐1** to **Se‐5** achieve significantly higher current densities as compared to measurements in *dry* electrolyte. At −1.8 V vs. NHE, which was identified as good starting point for CO_2_ electroreduction with **Pn**, the **Se‐Y** catalysts reach current densities between −25 and −30 mA cm^−2^ under *wet* conditions and −7 to −10 mA cm^−2^ under *dry* conditions, respectively. This behavior is attributed to the higher water content and the resulting increase in conductivity of the *wet* electrolyte. To achieve an elevated catalytic current in *wet* electrolyte, it can be noted that **Pn** (−1.50 V) and **Se‐1** (−1.57 V) require the most reducing potentials to reach a current density of −20 mA cm^−2^ (Figure 1 A). With increasing Se content, the required overpotential shifts to less positive values. The lowest value was reached for **Se‐4** with −1.46 V to achieve −20 mA cm^−2^. However, further increase of the Se‐content (**Se‐5**) leads again to a higher potential (−1.52 V) to achieve the same current density. As expected from previous HER experiments,^17^ our pentlandite analogues undergo substantial activation under reductive conditions resulting in higher current densities at comparable potentials after 2 hours of electrolysis. This phenomenon is most obvious for **Pn** (Δ*η*=140 mV) and **Se‐1** (Δ*η*=110 mV) possessing high sulfur content. The catalysts **Se‐2** to **Se‐5** undergo less pronounced activation between Δ*η*=50 mV and 80 mV (Figure 1 C), a phenomenon that can potentially be attributed to depletion of sulfur from the catalytic surface as we previously showed by XPS^9^ and operando phonon studies.^17^ Similar surface alterations were shown to be a decisive factor for the activation of (Se‐)pentlandites and surface conditioning under reductive HER conditions.^15^ Interestingly, upon transition to *dry* electrolyte conditions, the same catalysts undergo substantial deactivation under reductive potentials obvious by a shift of the potentials to more negative values. **Se‐1** experiences the highest degree of deactivation (Δ*η*=250 mV), whereas **Se‐3** seems to be the least affected catalyst (Δ*η*=90 mV) (Figure 1 F). For CO_2_RR investigation, solely initial information on the activity of the catalysts can be deduced from linear sweep voltammetry. It is evident that multiple competing reactions can occur during the reductive sweep. For example, a significant part of the observed current density could be a result of dihydrogen production, which is a commonly observed side reaction for CO_2_RR. Therefore, a thorough quantification of the generated products under electrocatalytic conditions is of utmost importance. Experiments were conducted at a constant potential of −1.8 V vs. NHE in a continuously CO_2_ purged saturated electrolyte which is, based on our previous reports,^9^ expected to yield a significant CO_2_RR activity for pentlandites while ensuring sufficient stability of the pellet electrode. Gaseous products were analyzed via online gas chromatography, while quantification of liquid samples was performed through an offline GC‐MS apparatus equipped with an evaporator unit. As no significant traces of liquid products such as alcohols, carboxylic acids or aldehydes were detected, further discussions of liquid products will be omitted.

**Figure 2:**
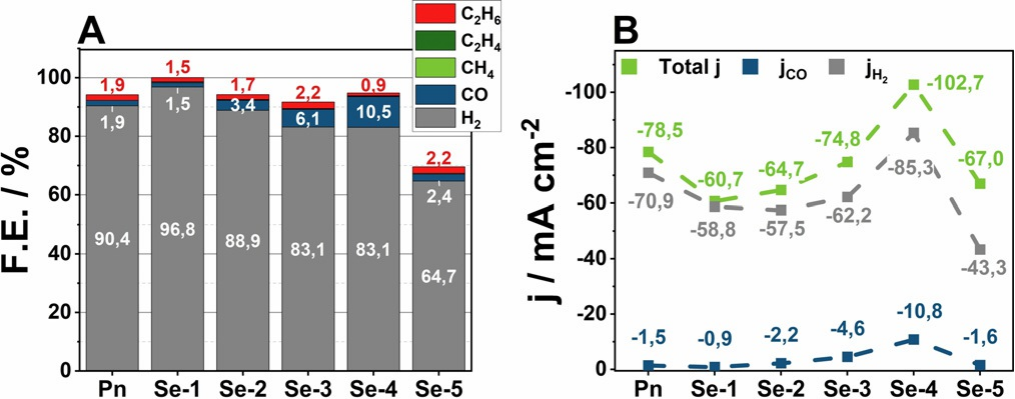
Faradaic efficiency of **Pn** to **Se‐5** at −1.8 V vs. NHE, in *wet* electrolyte acquired after 2 hours of electrolysis (A). Current density of **Pn** to **Se‐5** in *wet* electrolyte acquired after 2 hours of electrolysis (B).

Using *wet* electrolyte (30 000 ppm H_2_O), HER constitutes the dominant electrocatalytic reaction (Figure 2 A). While CO_2_RR under these conditions is suppressed, the main CO_2_RR products observed in the headspace gas mixture were CO (1050 ppm), and traces of hydrocarbons (CH_4_ (5 ppm), C_2_H_4_ (1 ppm)). Furthermore, ethane was detected in the gas phase (22 ppm). Notably, in terms of product selectivity, the ratio of H_2_/CO shifts in favor of carbon monoxide formation upon increasing the amount of Se in the respective **Se‐Y** catalysts. In particular, while **Se‐2** exhibits a F.E. of 3.4 % for CO and 88 % for H_2_, the F.E. for CO increases to 6.1 % for **Se‐3** concomitant with a decreased F.E. for H_2_ at 83 %. The increasing selectivity for CO reaches its maximum at **Se‐4** with a F.E. of 11 % for CO, accompanied by a F.E. for H_2_ of 83 %. Interestingly, **Se‐1** and **Se‐5** diverge from the described trend, with the former demonstrating an increased selectivity for HER and the latter a severely decreased F.E. of 65 %. The H_2_ selectivity of **Se‐1** is in accordance with previous investigations on this material showing an intrinsically increased HER activity compared to **Pn** and the other **Se‐Y** catalysts under acidic conditions.^15^ To understand the abrupt total activity decrease of **Se‐5**, the surface of the pellet electrode was examined by scanning electron microscopy (SEM) and energy dispersive X‐ray spectroscopy (EDX) after long‐term electrolysis under *wet* conditions. After 8 hours of electrolysis **Se‐5** possessed total F.E. of solely 70 %, consisting of 68 % H_2_ and 2 % CO at a constant current density of −55 mA cm^−2^ (Figure S12). While analysis of the pellet electrode after electrolysis did not reveal any changes in the stoichiometry, formation of a thick carbon film on the surface of the electrode (Figure S13) was observed. This film continuously lowers the overall activity of the catalyst towards CO and H_2_ formation and accounts for the overall lowered F.E. for both products via reduction of the adsorbed CO_2_ to carbon.

The overall highest current density was obtained for **Se‐4** (−102.7 mA cm^−2^) and significantly exceeds that of all other investigated compounds (Figure 2 B). At the same potential, the Se‐free **Pn** only possesses a current density of −78.5 mA cm^−2^. The seleno‐pentlandites **Se‐1** (−60.7 mA cm^−2^), **Se‐2** (−64.5 mA cm^−2^) and **Se‐3** (−74.8 mA cm^−2^) show even lower current densities compared to **Se‐4** and **Pn**. Importantly, the partial current density for CO (*j*_CO_) of the sulfoselenides **Se‐2** to **Se‐4** increases with the Se‐content, possessing a higher *j*_CO_ value for CO for each equivalent Se added and results in an apparent five‐fold current increase for **Se‐4**. Regarding **Se‐1**, the low current density for the CO_2_RR (−0.9 mA cm^−2^) is a result of its high selectivity for the competing HER, severely hindering its CO_2_ reducing activity.

Furthermore, to verify the origin of these increased activity trends, the electrochemical surface area (ECSA) of the investigated seleno‐pentlandites was determined from the electrochemical double‐layer capacitance (C_dl_) through cyclic voltammetry. Notably, the substitution of sulfur for selenium in the Fe/Ni‐pentlandite lattice results in insignificant changes to the ECSA, ranging from 0.0054 mF cm^−2^ (**Se‐5**) to 0.0071 mF cm^−2^ (**Se‐3**), across the investigated catalysts. This minimal effect of the S/Se exchange on the ECSA is also in good accordance to comparable CO_2_RR investigations employing sulfoselenides and suggests a similar electrode surface area.^13^, ^18^ During electrolysis, the ECSA of the investigated electrode increases for all compounds. The effect is most pronounced for electrodes containing high amounts of Se. Accordingly, as the S/Se exchange proceeds, **Pn** possesses the lowest post‐controlled potential coulometry (CPC) ECSA and **Se‐4** the highest ECSA with values of 0.0147 mF cm^−2^ and 0.0251 mF cm^−2^, respectively (Figure S10). This increase of the ECSA could be the result of enhancement of surface roughness of the electrode due to electrolysis. However, in this case, a steadily rising catalytic current should be observed as the continuously roughened electrode increases its surface area. Thus, this hypothesis is not supported by the respective current curves, which rather reveal a steady‐state character. In addition, post ex situ investigations of **Se‐4** after electrolysis revealed an unaltered flat and polished surface with an unaffected surface composition showing only a slightly lower S content (Table S3). We therefore attribute the observed increase of ECSA to the activation of the catalyst via sulfur‐depletion from the catalytic surface, a phenomenon previously observed for the Se‐free pentlandite and the seleno‐analogues under electrolytic conditions.^15^, ^19^

In order to further suppress the parasitic HER and study the effect of the S/Se exchange on **Pn**, we additionally performed electrolytic experiments at a constant potential of −1.8 V vs. NHE employing electrolytes with minimal amounts of water (24 ppm). This route should favor the CO_2_RR over the competing HER with CO being the main product and display conditions usually observed within sophisticated gas diffusion electrodes.^20^ Under such conditions all electrocatalysts show a substantially increased F.E. for CO, with a total F.E. close to 90 %. The remaining unquantified percentages could be attributed to the decomposition of acetonitrile, which is in line with previous reports.^21^ Notably, no other liquid products were detected under these conditions.

**Figure 3:**
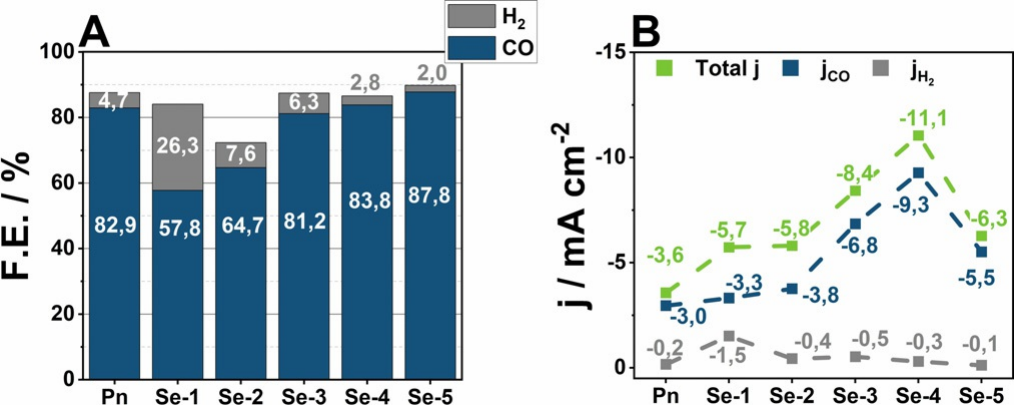
Faradaic efficiency of **Pn** ‐**Se‐5** at −1.8 V vs. NHE, in the *dry* electrolyte acquired after 2 hours of electrolysis **(A)**. Current density of **Pn** to **Se‐5** in the *dry* electrolyte acquired after 2 hours of electrolysis **(B)**.

It is worth mentioning that the tested **Se‐Y** catalysts retained a high selectivity for CO formation. Here, **Se‐4** and the, under *wet* conditions inactive, compound **Se‐5** possess a selectivity of 83.8 % and 87.8 % for CO, respectively. Similar values were obtained for **Se‐3** possessing a F.E. of 81.2 % (Figure 3 A). Furthermore, the decreasing selectivity of the selenium‐rich catalysts for HER as compared to their sulfur‐rich counterparts is also observed under these conditions with **Se‐4** and **Se‐5**, showing a F.E. for H_2_ of only 2.8 % and 2.0 %, respectively, compared to **Pn** (4.7 %). Interestingly, despite the low proton concentration, **Se‐1** and **Se‐2** retain a comparably high selectivity towards generation of hydrogen with F.E.s of 26.3 % and 7.6 %, respectively, along with an F.E. for CO of 57.8 % (**Se‐1**) and 64.7 % (**Se‐2**).

Subsequently, the catalytic stability of **Se‐5** was tested by long‐term electrolysis in *dry* electrolyte and revealed a constant CO production of 90 % at a current density of −9 mA cm^−2^ over 8 hours of electrolysis. Contrary to our expectations, investigation of the pellet electrode post electrolysis by SEM‐EDX measurements notably did not reveal increased carbon film formation as was observed in the respective case of the *wet* electrolyte (Figure S20). This altered behavior could indicate that the increased concentration of water negatively affects the catalytic properties of **Se‐5** and favors the hydrogenation of the adsorbed CO to carbon on the surface on the electrode similar to earlier reports by Han and co‐workers.^22^

The beneficial effect of the S/Se exchange can be even better observed by comparison of the respective current densities of the materials (Figure 3 B) with the **Se‐Y** compounds enabling higher current densities compared to **Pn** at a given potential. Likewise, during electrolysis the observed currents of all tested electrocatalysts steadily increase, a behavior that we attribute to the enrichment of water in our setup during electrolysis (Table S1) with the final H_2_O concentration after 2 hours of electrolysis being close to 150 ppm. While pertaining proton‐poor conditions, the increased water concentration in the catholyte most‐likely stems from water cross‐over from the anolyte that is constantly flushed with wet N_2_.

The partial CO current density substantially increases from **Pn** (−3.0 mA cm^−2^) to **Se‐4** (−9.3 mA cm^−2^), while for **Se‐5** it drops to −5.5 mA cm^−2^. Upon comparison of the partial current densities, **Se‐4** demonstrates the highest *j_CO_* values of −9.3 mA cm^−2^, followed by **Se‐3** and **Se‐5** with −6.8 mA cm^−2^ and −5.5 mA cm^−2^, respectively. Likewise, the sulfur‐rich variants **Pn** and **Se‐1** show a decreased CO_2_RR activity with a current density of −3.0 mA cm^−2^ and −3.3 mA cm^−2^.

In addition, significant changes of the ECSA can be observed after the electrolytic experiment (Figure S12). Here, the observed ECSA values during electrolysis vary in a non‐specific manner, with **Se‐1** (0.00736 mF cm^−2^) showing the highest increase compared to prior to electrolysis values.

Although current research on electrocatalysis focuses on potentiostatic experiments to evaluate the catalytic activity of materials, industrial electrolyzers commonly operate under galvanostatic conditions and significantly increased current densities, employing highly selective, efficient but also economically uncompetitive catalysts.^23^ For this reason, the most active CO_2_RR catalyst, **Se‐4**, was investigated at different current densities in different electrolytes (1 m KOH, 1 m KHCO_3_, *wet*, *dry*, Figure 5).

**Figure 4:**
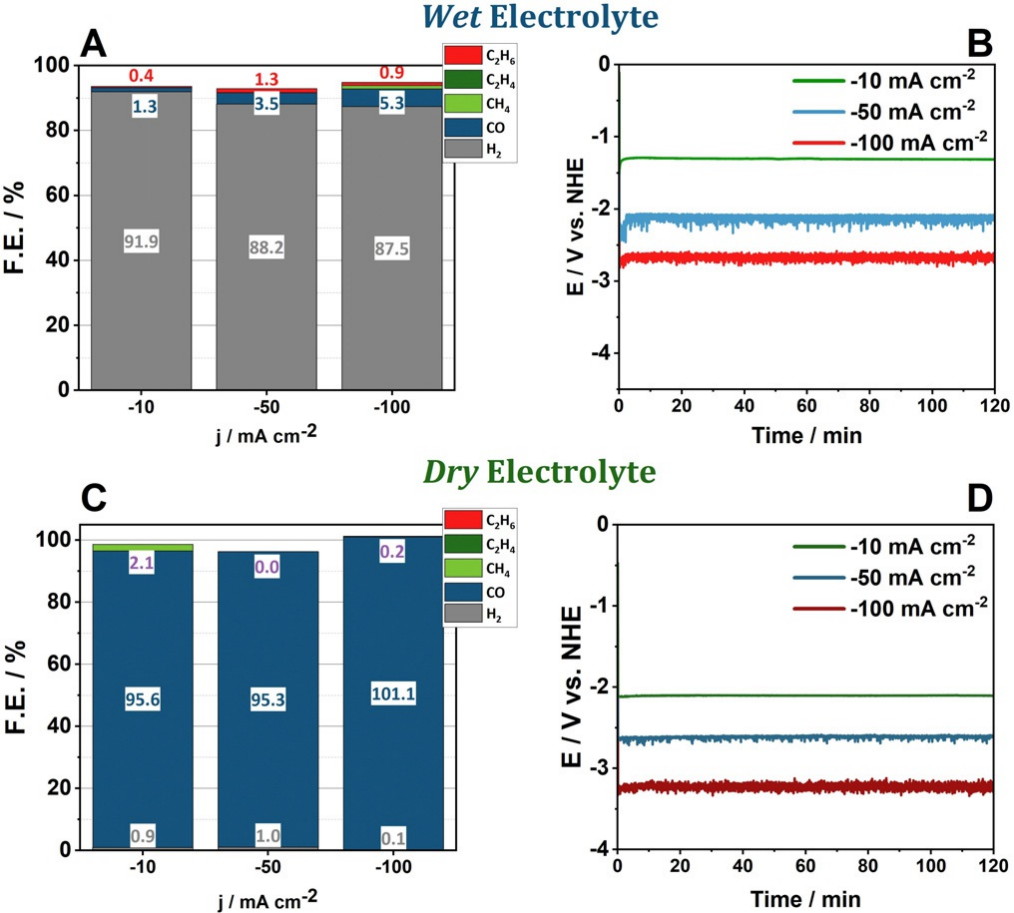
Faradaic efficiency of **Se‐4** in the CO_2_ saturated *wet* electrolyte across the applied current densities after 2 hours of electrolysis (A). Required potential to reach the applied current density in *wet* electrolyte (B). Faradaic efficiency of **Se‐4** in the CO_2_ saturated *dry* electrolyte across the applied current densities after 2 hours of electrolysis (C). Required potential to reach the applied current density in *dry* electrolyte (D).

Obviously, HER remains the dominant reaction across the applied current densities in *wet* electrolyte (Figure 4). Applying a current density of −10 mA cm^−2 (−^1.3 V vs. NHE), a F.E. of 91.6 % is observed for H_2_ development, decreases to 88.2 % for −50 mA cm^−2^ (−2.1 V vs. NHE) and ultimately reaches a value of 87.5 % for −100 mA cm^−2^ (−2.6 vs. NHE). Simultaneously, this decreased F.E. of the HER goes along with an increased CO_2_RR activity. Here, **Se‐4** possesses selectivities for CO of 1.3 % (−10 mA cm^−2^), 3.5 % (−50 mA cm^−2^) and 5.3 % (−100 mA cm^−2^). Notably, alongside with formation of CO, hydrogenation of acetonitrile to ethane takes place with an F.E. of 0.4 % (−10 mA cm^−2^), 1.3 % (−50 mA cm^−2^) and 0.9 % (−100 mA cm^−2^).

In contrast, galvanostatic experiments performed under proton‐poor conditions exhibit a significantly elevated CO production. For −10 mA cm^−2^ (−2.1 V vs. NHE), CO is the main product of the reduction reaction reaching an F.E. of 95.6 %, followed by an increased selectivity for CH_4_ formation, at an F.E. of 2.1 % with H_2_ possessing a value of 0.9 %.

Our previous experiments have demonstrated that CH_4_ and C_2_H_4_ stem from the reduction of CO_2_ and the emergence of ethane can be attributed to the hydrogenation of acetonitrile, as was demonstrated in our previous experiments employing ^13^CO_2_ and deuterated acetonitrile.^9^ We thus herein reinvestigated the product origins for **Se‐4** utilizing deuterated acetonitrile as the electrolyte (Figure S21). Here, GC‐MS analysis clearly shows that CH_4_ is a side product of the CO_2_ reduction and ethane stems from the decomposition of acetonitrile. Further increase of the applied current density to −50 mA cm^−2^ (−2.6 V vs. NHE) leads to complete suppression of CH_4_ formation and an F.E. for hydrogen formation of only 1.0 %, while the CO formation is practically unaltered (F.E. 95.3 %). At −100 mA cm^−2^ (−3.2 V vs. NHE) the product gas stream only consists of CO, reaching a quantitative F.E. Although the transition from *wet* to *dry* acetonitrile is accompanied by a potential increase of 0.5 V to 0.7 V, this additional potential input is outbalanced by the significantly increased selectivity for CO production.

**Figure 5:**
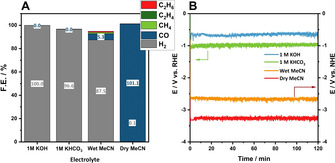
Faradaic efficiency of **Se‐4** in the employed CO_2_ saturated electrolytes at an applied current density of −100 mA cm^−2^ after 2 hours of electrolysis (A). Required potential to reach the applied current density, normalized to RHE (aqueous electrolyte) or NHE (organic electrolyte) (B).

In addition, we performed control experiments by employing a glassy‐carbon (GC) electrode as the working electrode at −100 mA cm^−2^ under *wet* and *dry* conditions to assess the advantage of our catalysts compared to otherwise inert electrode materials. Under *wet* conditions, the GC‐electrode required a potential of ca. ‐3.9 V vs. NHE to reach the target current density, generating mainly H_2_ at an F.E. of 94 % and CO with an F.E. of 0.2 % (Figure S22). Under *dry* conditions, employing glassy‐carbon as the working electrode leads to a substantial decomposition of the electrolyte, when −100 mA cm^−2^ are applied (−4 V vs. NHE), with no gaseous products being detected (Figure S23). Coupled with the aforementioned results, **Se‐4** demonstrates an improved efficiency and increased selectivity for the CO_2_RR. Furthermore, it maintains at constant potential over 8 h of electrolysis under galvanostatic conditions with CO being the major CO_2_RR product in *wet* (Figure S24) and *dry* electrolyte (Figure S25) suggesting a good catalyst stability. Analysis of fluid products post electrolysis under galvanostatic conditions did not reveal the generation of any further CO_2_RR products or possible side‐products commonly generated by the decomposition or hydrogenation of the employed solvent.^25^ Though the observed activity is on par with other electrodes under comparable conditions (Table S6),^9^, ^26^ organic electrolyte‐based electrolyzer systems have not been employed beyond the laboratory scale. Industrial CO_2_ prototype electrolyzers typically employ aqueous, and significantly more conductive electrolytes, mainly consisting of KOH or KHCO_3_.^10^, ^27^ We therefore performed galvanostatic experiments applying a current density of −100 mA cm^−2^ (Figure 5). Under such conditions no CO_2_ reduction products were detected, and hydrogen was formed with an F.E. of 100 % at a potential of −0.6 V vs. RHE and −1.0 V vs. RHE for 1 m KOH and 1 m KHCO_3_, respectively.

The diminished CO_2_RR activity of **Se‐4** in aqueous electrolytes is not only a result of the bifunctional properties of the material for HER and CO_2_RR but can also be attributed to the decreased solubility and subsequent decreased mass transport of CO_2_ in aqueous electrolytes. This difference therefore underlines the importance of tuning the electrolyte to favor a desired reaction in the case of bifunctional catalysts.

Furthermore, important implications are derived from the materials compositions in conjunction with their performance. Pentlandites with higher selenium content exhibit an increased activity and selectivity for the CO_2_RR compared to the reference material **Pn** under these CO_2_RR‐favoring conditions. Likewise, the underlying reaction kinetics of the HER is diametrically affected by geometric and electronic alterations caused by variation of interatomic distances at the active sites as demonstrated previously.^15^ In short, the average interatomic distances at the active sites and consequently also the dimensions of the underlying crystal lattice gradually increase with increasing Se content and with it the metal‐metal distances. This increase influences the binding strength of potential substrates as well as the formation of products. While short metal‐metal distances favor the binding of protons, increase of this distance seems to suppress this binding and instead favors CO_2_ binding. Simultaneously, it may be hypothesized that the increasing presence of selenium near the active sites promotes the CO_2_RR which reaches an optimum at an equal incorporation of the respective chalcogens (**Se‐4**), before severely declining for **Se‐5**_._

## Conclusions

In summary, we have demonstrated the beneficial effect of S/Se exchange on the CO_2_RR activity of pentlandites (Fe_4.5_Ni_4.5_S_8‐Y_Se_Y_ (Y=1–5)). Substituting sulfur with its homolog selenium leads to a major increase of the CO_2_RR activity with Fe_4.5_Ni_4.5_S_4_Se_4_, reaching a maximum selectivity for CO of 84 % at a current density of −11 mA cm^−2^ in acetonitrile. Depending on the exact stoichiometric composition as well as the proton concentration in the electrolyte, the presented catalysts can be understood either as bifunctional CO_2_RR/HER catalysts, leading to the formation of variable *syn*‐gas mixtures, or as efficient CO‐producing CO_2_RR catalysts.

The increased selectivity at higher Se content for CO‐production is further underlined by galvanostatic conditions at increased current densities approaching industrially relevant conditions (−100 mA cm^−2^), where especially Fe_4.5_Ni_4.5_S_4_Se_4_ showed quantitative conversion of CO_2_ to CO. Furthermore, our results reflect the structural and electronic changes induced in the pentlandite‐lattice through S/Se exchange and its CO_2_RR activity. While at low Se content HER is favored, high Se contents lead to facilitated CO_2_RR.

Notably, the catalysts can be applied in bulk form and no artificial nanostructuring is required, which underlines the simplicity of the herein described catalysts. In addition, the results show that both, the catalyst material as well as the reaction conditions, play a major role in tuning CO_2_RR activity. Thus, tuning of the reaction environment of the herein reported material class by altering electrode compositions should lead to enhanced CO_2_RR likewise in aqueous environments and is currently under investigation.

## Experimental Section

**Materials**: Unless otherwise stated, all chemicals were purchased from commercial vendors. Iron (Sigma–Aldrich, 99.99 %), Nickel (Riedel‐de Haen, 99.8 %), Sulfur (Sigma–Aldrich, 99.5–100.5 %), Selenium (Sigma–Aldrich, 99.999 %) were used without any further purification. HPLC‐grade acetonitrile (Fischer Scientifc) was used and diluted with deionized water purified by Millipore Direct‐Q purification system.

**Synthesis and characterization**: Synthesis of the Se‐free **Pn** and its seleno‐analogues was performed in similar fashion to our previous reports.^15^, ^16^ Powder X‐ray diffraction measurements were performed at a HUBER powder X‐ray diffractometer equipped with a Mo‐K_α_ source, *λ*=0.7093 Å. To ensure comparability of the results with literature, the obtained 2θ‐values were converted to the values resulting from a Cu K_α_ source (*λ*=1.5418 Å) according to Braggs’ law. For the differential scanning calorimetry measurements, a NETZSCH STA 449F3 apparatus was employed. Herein, the powder sample was homogenously distributed on the bottom of an aluminium oxide sample holder. Under a nitrogen (N_2_) atmosphere, approximately 50 mg of the sample substance was heated from 27 °C up to 1000 °C at a heating rate of 10 K min^−1^. Stability of the resulting phase transitions was assessed by cooling the sample at a rate of 10 K min^−1^ after reaching the maximal temperature. Scanning electron microscopy and electron X‐ray spectroscopy measurements were performed at a remX GmBH JEOL JSM‐6510 apparatus coupled with an EDAX Generis 4000 respectively. The powdered and pelletized samples were attached to a graphite sheet and studied under vacuum. Topological information was acquired by applying an acceleration voltage of 5 keV. The EDX measurements were performed though irradiation of the sample with an acceleration of voltage in the 0–20 keV region. Qualification of the elemental composition was performed through an internal database, allowing for the quantification of each element in the sample.

**Electrolysis experiments**: Fabrication of the working pellet electrode was performed according to previous reports.^28^ Electrochemical investigations were conducted using a GAMRY Reference 600 potentiostat and a three‐electrode setup, using the prepared iron‐nickel sulfoselenide electrodes as working electrode, Ag/AgCl (0.1 m TBAPF_6_, 0.1 m TBACl, 30 mg mL^−1^ H_2_O) as reference and a Pt‐mesh as counter electrode, with the half‐cell compartments being separated by a Fumasep F‐10 120P membrane. As supporting electrolyte 0.1 m TBAPF_6_ was used. The electrolyte solutions were continuously purged with CO_2_ at a constant rate of 10 mL min^−1^. For compensation of the iR drop between the reference and the working electrode, the embedded iR drop correction of the potentiostat was used. Prior to the desired electrochemical measurements, the electrolyte solution was saturated with CO_2_ by purging with the gaseous substrate for 30 min at a flow rate of 10 mL min^−1^. Electrode conditioning was achieved by cyclic voltammetry (35 cycles, 100 mV s^−1^) in the potential range of 0 V to −1.5 mV vs. NHE until a stable cyclic voltammogram shape was obtained. Linear sweep voltammetry was performed in the range of 0 to −2.1 V vs. NHE at a scan rate of 5 mV s^−1^, while the respective electrolyte solution was continuously stirred, and CO_2_ was bubbled through. The electrochemical surface area was subsequently determined by scanning in a catalytically non‐active range between −0.5 to −0.6 V vs. NHE at increasing scan rates of 10, 20, 30, 40, 50, 60 mV s^−1^. All potentiostatic measurements were performed at a constant potential of −1.8 V vs. NHE. Galvanostatic measurements were performed by applying the required current relative to the geometric area of the electrode (0.071 cm^2^) to obtain the target current density. To minimize the decomposition of acetonitrile on the anode when employing electrolytes with low proton availability, the anolyte was continuously purged with wetted N_2_ at a flow rate of 10 mL min^−1^ during electrolysis.

Quantification of the headspace gas composition was performed using an Agilent Technologies 7820A gas chromatograph equipped with a thermal conductivity (TCD) and a flame ionization detector (FID) as well as a methanizer. The gaseous mixture was separated using a two‐column separation system (HP‐PLOT Q column 30 m *x* 0.53 mm *x* 40 μm & HP‐Molesieve column 5 Å 30 m *x* 0.53 mm *x* 25 μm) with argon as the carrier gas. Quantification of alcohols and acids was performed using a Shimadzu GC‐MS QP2020 equipped with a KS20 headspace sampler. The exact water concentration of the *dry* electrolyte before and after electrolysis was determined by coulometric titration via Karl Fischer titrator using a TitroLine 7500 KF trace.

## Conflict of interest

The authors declare no conflict of interest.

## Biographical Information

*Ulf received his Ph.D. from the Friedrich‐Schiller University Jena. After a postdoctoral stay at MIT (2011/2012), he started his independent career at the Ruhr University Bochum funded by the “Fonds der Chemischen Industrie” and the DFG as an Emmy Noether group leader. He holds a professorship at the Ruhr University Bochum since 2019 and is leading the department Think Tank/Electrosynthesis at Fraunhofer UMSICHT. His research interests are in the field of technical electrochemistry with a special emphasis on the electrochemical reduction of CO_2_ and protons and catalyst design*.

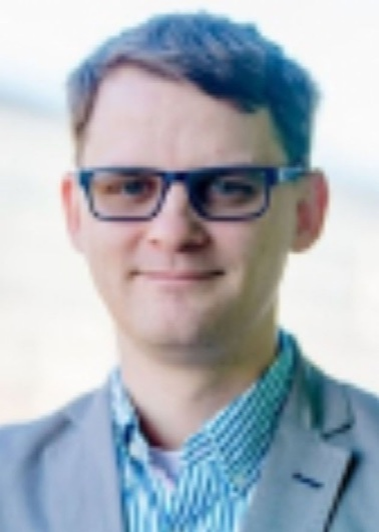

## Supporting information

As a service to our authors and readers, this journal provides supporting information supplied by the authors. Such materials are peer reviewed and may be re‐organized for online delivery, but are not copy‐edited or typeset. Technical support issues arising from supporting information (other than missing files) should be addressed to the authors.

Supplementary
