# Supplementary material for: Enhancing the CO2 Electroreduction of Fe/Ni‐Pentlandite Catalysts by S/Se Exchange
Source: Chemistry. 2020 Jul 8;26(44):9938–44. doi: 10.1002/chem.202001289 (PMC7496145; doi:10.1002/chem.202001289)
Supplement: Supplementary file 1 — Supplementary [file CHEM-26-9938-s001.pdf]

Author Contributions

K.P. Data curation: Equal; Investigation: Equal; Writing - Original Draft: Equal
M.S. Data curation: Equal; Investigation: Equal; Writing - Original Draft: Equal
D.S. Validation: Equal; Writing - Original Draft: Equal; Writing - Review & Editing: Equal
U.A. Conceptualization: Lead; Formal analysis: Supporting; Funding acquisition: Lead; Investigation: Supporting; Methodology: Lead; Project administration: Lead; Supervision: Lead; Writing - Original Draft: Supporting; Writing - Review & Editing: Lead.
